# Severe Hyperlipidemia and Multifocal Atherosclerosis With Negative Genetic Testing for Familial Hypercholesterolemia: A Case Report

**DOI:** 10.7759/cureus.110527

**Published:** 2026-06-09

**Authors:** Dawid Buczyński, Anna Róg, Mateusz Pałka, Michał Susuł, Renata Rajtar-Salwa

**Affiliations:** 1 Students' Scientific Group at the 1st Department of Cardiology, Interventional Electrocardiology and Arterial Hypertension, Jagiellonian University Medical College, Kraków, POL; 2 Faculty of Medicine, Jagiellonian University Medical College, Kraków, POL; 3 Clinical Department of Cardiology and Cardiovascular Interventions, University Hospital, Kraków, POL; 4 2nd Department of Cardiology, Institute of Cardiology, Jagiellonian University Medical College, Kraków, POL

**Keywords:** celsr2 gene, familial hypercholesterolemia, lipoprotein(a), low-density lipoprotein cholesterol, variant of uncertain significance

## Abstract

Familial hypercholesterolemia (FH) is a genetic disorder characterized by severely elevated cholesterol levels and increased cardiovascular risk. While typically associated with mutations in the low-density lipoprotein receptor (*LDLR*), apolipoprotein B (*APOB*), or proprotein convertase subtilisin/kexin type 9 (*PCSK9*) genes, many clinical cases remain genetically unexplained. Identifying novel variants in non-canonical genes is essential for understanding the complex genetic architecture of lipid disorders. A 75-year-old female patient presented with severe hyperlipidemia, multifocal atherosclerosis, and multiple comorbidities. Her native low-density lipoprotein cholesterol (LDL-C) was 6.7 mmol/L. Based on a Dutch Lipid Clinic Network (DLCN) score of 9 (5 points for LDL-C level, 2 points for personal premature coronary artery disease (CAD), and 2 points for a first-degree relative - specifically the patient's father - with xanthomata of the Achilles tendons area, with personal tendon xanthomata and corneal arcus being absent), a clinical diagnosis of definite familial hypercholesterolemia was established. Furthermore, an elevated lipoprotein(a) level of 407.5 nmol/L was identified as an independent cardiovascular risk factor. Comprehensive genetic testing revealed no pathogenic mutations in the *LDLR*, *APOB*, or *PCSK9* genes. However, molecular analysis identified a heterozygous variant of uncertain significance (VUS), c.7031G>T p.(Arg2344Leu), in the Cadherin EGF LAG Seven-Pass G-Type Receptor 2 (*CELSR2*) gene. This case demonstrates the diagnostic challenges in patients who fulfill the clinical criteria for familial hypercholesterolemia but lack mutations in primary candidate genes. Genetic testing revealed a rare missense VUS in the *CELSR2* gene. However, this finding should be interpreted as an incidental VUS of unproven clinical relevance, as no mechanistic link between this specific coding variant and the patient’s severe hyperlipidemic phenotype has been established. Further functional studies are required to determine the clinical significance of this specific *CELSR2 *variant in the context of cardiovascular risk.

## Introduction

Familial hypercholesterolemia (FH) is a common genetic disorder leading to premature atherosclerosis and high cardiovascular risk [1,2]. Clinical diagnosis relies on validated scoring systems and molecular testing of classical causative genes (low-density lipoprotein receptor (*LDLR*), apolipoprotein B (*APOB*), or proprotein convertase subtilisin/kexin type 9 (*PCSK9*)). However, many patients with a definitive FH phenotype lack identifiable mutations in these loci. These mutation-negative cases often represent "FH phenocopies" arising from a polygenic architecture or alternative genetic variants [3]. Nevertheless, the clinical relevance of rare coding variants within the Cadherin EGF LAG Seven-Pass G-Type Receptor 2 (*CELSR2*)gene remains poorly characterized.

Herein, we describe the clinical management of a 75-year-old female patient with phenotypic FH (Dutch Lipid Clinic Network (DLCN) score of 9) [1] and advanced multifocal atherosclerosis complicated by severe statin intolerance. Furthermore, we discuss the appropriate interpretation of a rare heterozygous *CELSR2* variant of uncertain significance (VUS) identified via next-generation sequencing (NGS), which - lacking functional or co-segregation data - must be evaluated strictly as an incidental finding of unproven relevance rather than a primary explanatory mechanism for the severe lipid phenotype.

## Case presentation

The patient, currently aged 75, had been under long-term cardiological care due to advanced, multifocal atherosclerotic disease. Her medical history (*CELSR2* variant, Table 1) revealed premature development of coronary artery disease; her first non-ST-elevation myocardial infarction (NSTEMI) occurred in the year 2000 at the age of 50. In subsequent years, multivessel atherosclerosis progressed aggressively, involving the coronary, carotid, and lower limb arteries, which necessitated multiple interventional treatments. These included a percutaneous coronary intervention (PCI) with a stent implanted in the left anterior descending (LAD) artery in 2007, right carotid artery stenting (CAS) in November 2017 due to critical stenosis (with concurrent chronic occlusion of the left internal carotid artery), and superficial femoral artery stenting with a drug-eluting stent (DES) in April 2018.

**Table 1:** Chronological timeline of the patient's case history.

Date/period	Clinical event/diagnostic and therapeutic episodes
2000	First non-ST-elevation myocardial infarction (NSTEMI) at age 50; treated conservatively. Type 2 diabetes mellitus diagnosed.
2007	Percutaneous coronary intervention (PCI) with stent implantation in the left anterior descending (LAD) artery.
November 2017	Carotid artery stenting (CAS) of the right common/internal carotid artery (RCCA/RICA) due to critical stenosis. Occlusion of the left internal carotid artery (LICA) documented.
April 2018	Stenting of the right superficial femoral artery with a drug-eluting stent (DES) due to severe peripheral artery disease.
May 2023	Statin-associated rhabdomyolysis and acute kidney injury (AKI) during high-dose rosuvastatin therapy (40 mg daily). Rosuvastatin immediately discontinued; acute clinical management initiated.
August 2023	Resolution of statin-associated muscle symptoms (SAMS) and normalization of muscle enzymes/renal parameters. Baseline parameters re-established.
October 2023	Careful, low-risk statin rechallenge initiated with low-dose pitavastatin under strict monthly creatine kinase (CK) monitoring.
January 2024	Suboptimal lipid control on pitavastatin (LDL-C = 3.31 mmol/L). Clinical phenotype scored 9 points on the Dutch Lipid Clinic Network (DLCN) scale ("definite FH"). Familial hypercholesterolemia suspected; blood drawn for next-generation sequencing (NGS). Regimen switched to a single-pill combination of rosuvastatin/ezetimibe (20/10 mg).
May 2024	Genetic report finalized: LDLR MLPA negative; NGS identified an incidental heterozygous CELSR2 variant of uncertain significance (VUS), c.7031G>T p.(Arg2344Leu).
September 2024	Patient evaluated and found to meet all structural and laboratory inclusion criteria for the national proprotein convertase subtilisin/kexin type 9 (PCSK9) inhibitor drug program.
March 19, 2025	Evolocumab (140 mg s.c. every 2 weeks) formally initiated as an essential adjunct to a low-dose tolerated statin/ezetimibe regimen (Mizetam 40/10 mg).
June 11, 2025	Three-month follow-up: target lipid control successfully achieved with an LDL-C reduction to 0.9 mmol/L; excellent clinical tolerance.
February 2026	Twelve-month follow-up: sustained long-term efficacy, safety, and stable muscle enzymes documented.

Comorbidities included type 2 diabetes mellitus (diagnosed in 2000), hypertension, and obesity (Body Mass Index (BMI) = 32.8 kg/m²). Long-term smoking (10 cigarettes per day for over 50 years) represented a major contributing cardiovascular risk factor.

Historically, laboratory tests prior to the initiation of intensive lipid-lowering therapy revealed a very high native low-density lipoprotein cholesterol (LDL-C) concentration, reaching 6.7 mmol/l, alongside significantly elevated baseline lipoprotein(a) levels (Table 2). In January 2024, a systematic clinical re-evaluation was performed. On the DLCN scale, the patient scored a total of 9 points, meeting the diagnostic criteria for definite familial hypercholesterolemia [1]. The itemised breakdown of the score was as follows: (i) Five points for a baseline LDL-C level of 6.7 mmol/L (range: 6.5-8.4 mmol/L), (ii) two points for personal premature coronary artery disease, and (iii) two points for a first-degree relative - specifically the patient's father - with xanthomata of the Achilles tendon area.

**Table 2:** Key laboratory findings before and during lipid-lowering therapy. * General reference range; individual target depends on cardiovascular risk [2]. ** High-density lipoprotein cholesterol (HDL-C) concentration represents a high-normal, cardiovascularly protective level and was not oriented in the atherogenic direction. *** Lp(a) mass was measured using an immunoturbidimetric assay. # General reference range for fasting glucose; therapeutic target for patients with diabetes is individualized. † General reference range; therapeutic target for patients with diabetes is typically ≤ 7.0%. ‡ Reference range for adult females. § Target LDL-C for patients at extreme cardiovascular risk according to European Society of Cardiology (ESC) guidelines [2,4].

Laboratory parameter	Result	Unit	Reference range
Baseline lipid profile			
Low-density lipoprotein cholesterol (LDL-C)	6.70	mmol/L	< 3.40 *
Total cholesterol (TC)	9.50	mmol/L	< 5.20
High-density lipoprotein cholesterol (HDL-C)	2.10**	mmol/L	> 1.29
Triglycerides (TG)	1.70	mmol/L	< 2.30
Lipoprotein(a)	407.50	nmol/L	< 125 nmol/L ***
Glycemic control			
Fasting plasma glucose (FPG)	129.60	mg/dL	70 - 99 #
Glycated hemoglobin (HbA1c)	6.70	%	4.30 - 5.90 †
Complications and follow-up			
Creatine kinase (CK) peak	6910	U/L	26 - 192 ‡
Achieved low-density lipoprotein cholesterol (LDL-C)	0.90	mmol/L	< 1.00 §

Physical examination revealed no personal tendon xanthomata or corneal arcus (0 points). Additionally, as a separate and independent biomarker of cardiovascular risk, a significantly elevated lipoprotein(a) concentration of 407.5 nmol/L was observed; notably, this metabolic parameter is not a component of the DLCN scoring system.

Genetic testing was performed to assess the molecular basis. Multiplex Ligation-dependent Probe Amplification (MLPA) analysis revealed no deletions or duplications in the *LDLR* gene. NGS failed to identify pathogenic variants in genes classically associated with monogenic familial hypercholesterolemia (*LDLR*, *APOB,* *PCSK9* genes) [5].

However, a heterozygous missense variant c.7031G>T p.(Arg2344Leu) was identified in exon 22 of the Cadherin EGF LAG Seven-Pass G-Type Receptor 2 (*CELSR2*) gene (rs70931149). To objectively evaluate this finding, detailed variant-level evidence was analyzed. This specific variant is extremely rare, with an allele frequency of 0.0000088 in the European population according to the gnomAD Exomes database, and it is currently not registered in major genomic databases, including ClinVar, Leiden Open Variation Database (LOVD), or The Human Gene Mutation Database (HGMD). In-silico functional prediction algorithms (via VarSome and Franklin) yielded highly discordant results, ranging from pathogenic to benign, resulting in an unresolved consensus prediction. Based on the American College of Medical Genetics and Genomics (ACMG) guidelines, the variant was classified as a variant of uncertain significance (VUS) [6].

Importantly, the clinical relevance of *CELSR2* alterations in the pathogenesis of familial hypercholesterolemia remains highly controversial. A recent targeted FH gene-panel study conducted at our institution by Totoń-Żurańska et al. evaluated mutation-negative Polish FH patients and explicitly included *CELSR2* in their analysis [7]. Their findings demonstrated that *CELSR2* variants did not constitute a primary molecular cause of FH within this population. The lack of prior literature, combined with the absence of co-segregation or functional data, firmly aligns with these local findings. Consequently, this *CELSR2 *variant must be interpreted strictly as an incidental VUS of unproven relevance, rather than a novel genetic locus explaining the patient's severe hyperlipidemic phenotype.

The patient showed limited tolerance to intensive statin therapy. In May 2023, while receiving intensive lipid-lowering therapy, she experienced an acute, severe episode of statin-associated muscle symptoms (SAMS) culminating in overt rhabdomyolysis. Crucially, the exposure preceding this event was high-dose rosuvastatin (40 mg daily); ezetimibe (10 mg daily), which was co-administered at the time, is not a recognized standalone cause of rhabdomyolysis, though the patient's advanced age, longstanding type 2 diabetes, and potential drug interactions likely amplified the rosuvastatin-associated myotoxicity. At the peak of the episode, laboratory findings revealed a severe elevation in creatine kinase (CK) activity reaching 6,910 U/L (approximately 35× the upper limit of normal) alongside marked mioglobinemia. Concurrently, a severe deterioration in renal function was documented, with serum creatinine peaking at 250 µmol/L, indicating acute kidney injury (AKI) secondary to myoglobinuria. High-dose rosuvastatin was immediately discontinued, and ezetimibe was temporarily held. Acute management consisted of prompt hospitalization, aggressive intravenous fluid resuscitation, and urinary alkalinization to maintain high urine output. This strategy successfully prevented permanent renal sequelae; by August 2023, the patient's muscle symptoms resolved, and both muscle enzymes and renal function completely normalized (CK = 67 U/L, creatinine = 89 µmol/L).

Given the patient’s extreme cardiovascular risk, history of myocardial infarction, and extensive vascular interventions, absolute discontinuation of lipid-lowering therapy was clinically unacceptable [8]. However, reintroducing any statin after an episode of clinical rhabdomyolysis with a CK elevation of this magnitude is a high-risk medical decision. To justify a safe statin rechallenge, a highly cautious and structured safety narrative was adopted under close clinical surveillance and strict monthly CK monitoring. In October 2023, the patient was initially rechallenged with low-dose pitavastatin (2 mg daily), selected specifically due to its minimal cytochrome P450-mediated drug interactions and favorable muscle safety profile. Although tolerated well without recurrence of SAMS, this low-intensity regimen provided insufficient lipid efficacy, with follow-up testing showing an LDL-C level remaining significantly elevated at 3.31 mmol/L. Consequently, in January 2024, a careful cross-titration was performed by transitioning the patient to a lower-dose intensity regimen of rosuvastatin using a single-pill combination of rosuvastatin/ezetimibe (initially 20/10 mg daily, later cautiously optimized to a tolerated maintenance dose of 40/10 mg daily). Under rigorous monthly laboratory surveillance, this reduced, moderate statin dose was exceptionally well tolerated, maintaining a stable baseline CK activity (59 U/L) without any recurrence of myalgia.

Due to the permanent inability to reach the strict guideline-recommended therapeutic targets using a restricted, lower-dose statin regimen alone, the patient was evaluated for advanced biologic therapies. On March 19, 2025, she was formally enrolled in the national drug program, and treatment with a PCSK9 inhibitor (evolocumab 140 mg every two weeks) was initiated as an essential adjunct to her tolerated low-dose rosuvastatin/ezetimibe therapy [9,10]. Following the addition of evolocumab, an optimal therapeutic response was rapidly documented. At her three-month follow-up on June 11, 2025, an LDL-C concentration of 0.9 mmol/L was successfully achieved, marking a profound improvement in lipid control without a single systemic or local adverse event. Long-term compliance and complete safety remained flawless through her most recent 12-month programmatic follow-up in February 2026.

## Discussion

The presented case highlights the inherent challenges and limitations of genetic diagnosis in severe lipid disorders. Despite a clinical phenotype definitively consistent with FH according to the DLCN scale (9 points) [1], the presence of a classic monogenic pathogenic variant in the *LDLR*, *APOB*, or *PCSK9* genes was not confirmed. In such mutation-negative scenarios, a polygenic etiology - characterized by an accumulation of common, small-effect LDL-C-raising alleles - is frequently suspected [3]. However, a formal LDL-C polygenic risk score was not computed for our patient due to the technical configuration of the utilized NGS diagnostic panel, which was optimized strictly for qualitative variant identification rather than polygenic burden quantification. Therefore, a polygenic basis remains a plausible but unproven hypothesis in this specific instance.

It is critical to conceptually separate this polygenic mechanism from the identification of the rare heterozygous *CELSR2 *variant. These represent distinct and competing genetic paradigms: a single rare coding variant of unknown function versus a cumulative global burden of common non-coding polymorphisms. As noted previously, the detected *CELSR2* coding sequence alteration is not a classic causative mutation for FH and must be categorized strictly as an incidental variant of uncertain significance (VUS) with no established mechanistic link to the patient's severe phenotype.

Beyond the unproven genetic architecture, the clinical presentation was heavily driven by the patient's extensive burden of classic, non-genetic cardiovascular risk factors, including a 50-year history of smoking, type 2 diabetes mellitus, and obesity. Rather than treating the incidental *CELSR2* VUS as a direct modifier, the severe, multifocal nature of the patient's atherosclerosis is more accurately interpreted as the synergistic result of an extreme clinical lipid phenotype interacting over decades with these profound metabolic and environmental risk factors.

Patient's perspective

From the patient’s perspective, decades of managing severe cholesterol and multifocal vascular disease had been a source of continuous anxiety. She recalled experiencing recurrent muscle cramps and abdominal discomfort during early treatment attempts, which severely affected her quality of life. The severe episode of rhabdomyolysis in May 2023 was particularly frightening, leaving her highly apprehensive about taking any cholesterol-lowering medications again. She noted that undergoing advanced genetic testing provided helpful clarity, reassuring her that her condition was being handled with maximum diagnostic precision. Following her enrollment in the biologic drug program in March 2025, she experienced an immense sense of relief. She found the bi-weekly injections straightforward to self-administer at home and reported being completely free of the previous debilitating muscle symptoms, expressing great satisfaction that her cholesterol levels had finally dropped to a safe range.

## Conclusions

A clinical diagnosis of definite FH based on high DLCN criteria warrants aggressive secondary prevention, even in the absence of an identifiable monogenic mutation. When high-dose statins are strictly precluded by severe adverse events such as rhabdomyolysis, the prompt initiation of a PCSK9 inhibitor (evolocumab) combined with low-dose, tolerated lipid-lowering therapies represents a safe and highly effective strategy to achieve stringent, guideline-recommended LDL-C targets.

Concurrently, in the era of widespread next-generation sequencing, the identification of rare variants in alternative loci, such as *CELSR2*, must be interpreted with extreme caution. In the absence of functional, population-specific, or co-segregation data, such genetic findings must be managed strictly as incidental VUS of unproven relevance, ensuring they do not detract from the management of the primary clinical phenotype or established cardiovascular risk factors.
